# Physical Therapist and Physical Therapist Student Knowledge, Confidence, Attitudes, and Beliefs About Providing Care for People With Dementia: A Mixed-Methods Systematic Review

**DOI:** 10.1093/ptj/pzac010

**Published:** 2022-02-12

**Authors:** Stephen M Quick, David A Snowdon, Katherine Lawler, Jennifer L McGinley, Sze-Ee Soh, Michele L Callisaya

**Affiliations:** 1 Peninsula Clinical School, Central Clinical School, Monash University, Melbourne, Australia; 2 Academic Unit, Peninsula Health, Melbourne, Australia; 3 Wicking Dementia Research and Education Centre, College of Health and Medicine, University of Tasmania, Hobart, Australia; 4 Department of Physiotherapy, University of Melbourne, Melbourne, Australia; 5 Department of Physiotherapy, Monash University, Melbourne, Australia; 6 Department of Epidemiology and Preventative Medicine, Monash University, Melbourne, Victoria, Australia; 7 Menzies Institute for Medical Research, College of Health and Medicine, University of Tasmania, Hobart, Australia

**Keywords:** Alzheimer Disease, Dementia, Education: Physical Therapist Students, Physical Therapists

## Abstract

**Objective:**

The purpose of this study was to determine physical therapists’ and physical therapist students’ attitudes and beliefs, knowledge, and confidence in working with people with dementia.

**Methods:**

This was a mixed-methods systematic review. Participants included physical therapists working in any clinical specialty and physical therapist students who had completed at least 1 clinical placement. Eleven databases were searched. The evidence was evaluated using the Joanna Briggs Institute Critical Appraisal Checklists. Data synthesis followed a convergent integrated approach according to Joanna Briggs Institute methodology for mixed-methods systematic reviews. Quantitative data were “qualitized” using thematic analysis and synthesized with qualitative data using thematic synthesis.

**Results:**

Fifteen studies were included (9 quantitative and 6 qualitative studies). Seven key themes evolved. Five related to the belief that (1) working with people with dementia is complex and challenging; (2) opportunities for education in dementia care are lacking; (3) working with people with dementia is a specialized area of practice; (4) there are unsupportive systems for working with people with dementia; and (5) people with dementia deserve rehabilitation, but their potential to improve is less certain. One theme related to knowledge (lack of knowledge in some areas of dementia care), and 1 theme related to confidence (lack of confidence in working with people with dementia).

**Conclusions:**

Physical therapists and physical therapist students believe that working with people with dementia can be challenging. The low levels of knowledge and confidence in areas important to working with people who have dementia suggest that more education about dementia is needed.

**Impact:**

This mixed-methods systematic review highlights that physical therapists and physical therapist students believe that working with people who have dementia is complex and challenging. Physical therapists want more training and support in this growing area of practice.

## Introduction

Dementia is a leading cause of disability.^1^ With approximately 50 million people living with dementia worldwide,^2^ dementia presents a major challenge to not only the individual but also health and aged care systems.^3^^,^^4^ With life expectancy rates rising in most parts of the world, it is estimated that the number of people with dementia will triple by 2050.^2^ Symptoms include impairments in cognition, behavior, physical (eg, balance and mobility), and psychological functioning.^5^ These symptoms can lead to an inability to perform activities of daily living as well as participate in social or life roles.

The clinical management of people with dementia, either as a primary diagnosis or as a co-morbidity, can be complex.^2^^,^^6^^,^^7^ Physical therapists play a key role in the care of people living with dementia, for example, after conditions such as a hip fracture, stroke, or a respiratory infection. Physical therapists also have a role in treating the symptoms of dementia. Exercise may be beneficial in reducing neuropsychiatric symptoms and cognitive decline,^8^ and multi-modal exercise programs longer than 3 months can improve functional strength, balance, mobility, endurance, and activities of daily living.^9–11^ With increasing evidence for physical therapy interventions in the care of people living with dementia, it is important that physical therapists and physical therapist students have adequate knowledge and confidence levels as well as attitudes and beliefs that facilitate effective and equitable care.

Low levels of dementia knowledge have been observed in health care workers,^12^^,^^13^ residential aged care staff,^13^^,^^14^ and undergraduate social workers.^15^ In some instances, health professionals have reported limited or no stand-alone dementia topics in their undergraduate curriculum.^13^ Where low knowledge and confidence levels in managing and caring for people with dementia is evident, health professionals also believed people with dementia had limited capacity to improve.^16^^,^^17^ In contrast, associations have been found between higher levels of education and better knowledge levels^12^ and between training and improved quality of care.^12^^,^^14–20^ With education and training opportunities perceived as an unmet need by other professions,^12^^,^^18^^,^^19^ it is important to understand this within the physical therapy context.

Given the prominent role for physical therapists in clinical care for people with dementia, it is important to understand both physical therapists’ and physical therapist students’ attitudes and beliefs, knowledge, and confidence about working with people with dementia. A better understanding of these issues will inform undergraduate, post-graduate, and on-the-job education and training. The research questions for this review were:

1. What are physical therapists’ and physical therapist students’ attitudes and beliefs on working with people with dementia?
2. How knowledgeable and confident are physical therapists and physical therapist students in working with people with dementia?

## Methods

This mixed-methods systematic review was prospectively registered in PROSPERO (CRD42020181845) and the protocol published.^21^

### Data Sources and Searches

Eleven electronic databases, including: Ovid MEDLINE, Cumulative Index of Nursing and Allied Health Literature, Ovid Embase, Emcare, PsycINFO, Scopus, Web of Science, Informit, Proquest Dissertations, Education Resources Information Centre, and Google Scholar were searched for thoroughness, with no date restrictions on January 2, 2021. Search terms were discussed and agreed on by the research team. The population of interest (ie, physical therapists and physical therapist students), context (ie, care of a person with dementia in any setting or country), and phenomena of interest (ie, attitudes, beliefs, knowledge, confidence) were linked using the “and” operator and within concept terms with the “or” operator. Broader search terms were used for the phenomena of interest (ie, opinions, experiences, reflections, education, expertise, values, perceptions, barriers, facilitators) to ensure all studies with data relevant to the review aims were captured. A detailed search strategy is presented in Supplementary Appendix 1. A search of the references and citations of eligible full-text articles was also undertaken.

### Study Selection

Qualitative and quantitative studies were included if they met the predetermined inclusion criteria. Two reviewers (S.Q. and D.S.) independently screened titles and abstracts using Covidence (Veritas Health Innovation, Melbourne, Australia). Three reviewers (S.Q., D.S., and M.C.) contributed to the independent double screening of full-text articles to assess for eligibility. Any differences of opinion among the reviewers were resolved by consensus.

To be included in this review, quantitative or qualitative studies needed to investigate physical therapists’ or physical therapist students’ attitudes (a feeling or opinion about something or someone, or a way of behaving that is caused by this),^22^ beliefs (the feeling of being certain that something exists or is true),^22^ knowledge (a skill in, understanding of, or information about something, which a person gets by experience or study),^22^ or confidence (a feeling that you can trust someone or something to work well or behave as you expect)^22^ on working with people with dementia. Separating the terms “attitudes” and “beliefs” is difficult, and past studies have incorporated these phenomena together.^23^^,^^24^ This is due to some definitions of the term “belief” incorporating the term “attitude.”^25–27^ The Cambridge Dictionary of Sociology^27^ defines attitudes as being “variously defined as an orientation (towards a person, situation, institution, or social process) that is held to be indicative of an underlying value or belief, or, among those who insist that attitudes can only be inferred from observed behavior, as a tendency to act in a certain (more or less consistent) way towards persons and situations.” Therefore, for the purposes of this review, the terms “attitudes” and “beliefs” were kept together for simplicity. Because the term “belief” precedes the term “attitudes,” it will be used to describe both phenomena in the results of this review*.* Studies could investigate attitudes, beliefs, knowledge, or confidence on working with people with dementia in any setting (eg, hospital, private clinic, outpatient, or aged care setting) or country. If studies investigated physical therapists’ and physical therapist students’ attitudes, beliefs, knowledge, or confidence on working with a general population of older adults, they were excluded. Studies including physical therapists working in any clinical specialty (eg, gerontology, orthopedic, neurological, cardio-respiratory) and of any experience were eligible. Physical therapist students must have completed at least 1 clinical placement. Studies that included other professions were included if findings specific to physical therapists or physical therapist students could be extracted. Studies that explored other phenomena of interest (ie, opinions, experiences, reflections, education, expertise, values, perceptions, barriers, facilitators) were included if data related to physical therapists’ or physical therapist students’ knowledge, confidence, or beliefs about dementia care could be extracted. For qualitative studies, the themes or quotes extracted had to be attributable to the physical therapy profession.

### Data Extraction and Quality Assessment

The quantitative studies (and quantitative component of mixed-methods studies) that were retrieved were assessed by 2 reviewers (S.Q. and M.C.) for methodological quality using the 8-item Joanna Briggs Institute Critical Appraisal Checklist for Cross-Sectional Studies.^28^ The qualitative studies (and qualitative component of mixed-methods studies) were also assessed by 2 reviewers (S.Q. and K.L.), using the 10-item Joanna Briggs Institute Critical Appraisal Checklist for Qualitative Research for qualitative studies.^28^ Items not applicable were not taken into consideration on the overall grading of each study. Any disagreements between reviewers, including items deemed “unclear” or “not applicable,” were resolved through discussion. Where a study recorded a “yes” for the majority (>50%) of applicable questions, it was deemed to be of high quality. All studies underwent data extraction regardless of the results of their methodological quality.

Quantitative and qualitative data were extracted from studies included in the review independently by 2 reviewers using the Joanna Briggs Institute Mixed Methods Data Extraction Form.^28^ Information pertaining to the population (eg, physical therapist or student, age, specialty, and experience), study design (qualitative or quantitative), phenomena of interest measurement tool (eg, dementia knowledge assessment tool), and context (care of a person with dementia in any setting or country) were summarized. For quantitative studies, data extracted included descriptive data or inferential statistics. For intervention studies, only pre-intervention data were extracted. For qualitative studies, information included results of the studies verbatim, including themes and subthemes with corresponding quotations from participants.

### Data Synthesis and Analysis

#### Overview

**Figure 1:**
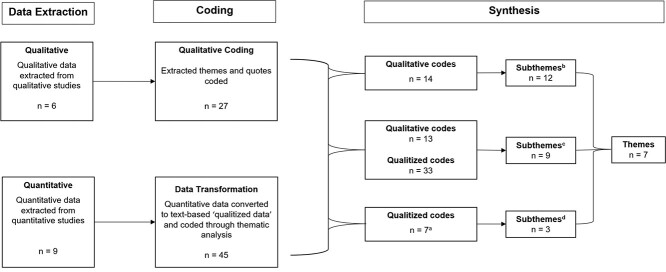
Thematic synthesis using a convergent integrated approach.^28^ n = the number of studies, codes, sub-themes and themes that were used or created at each step of the process of the review. ^**a**^5 qualitized codes did not contribute to the creation of sub-themes/themes. ^**b**^12 subthemes were created from synthesis of 14 qualitative codes. ^**c**^9 subthemes were created from synthesis of 13 qualitative codes and 33 qualitized codes. ^**d**^3 subthemes were created from synthesis of 7 qualitized codes only.

This was a mixed-methods systematic review, which combines different methods (such as quantitative and qualitative) to answer a research question. Data analysis followed a convergent integrated approach according to the Joanna Briggs Institute methodology for mixed-methods systematic reviews.^28^ The approach allows for the analysis of quantitative and qualitative data at the same time. The approach involved 2 steps: (1) the transformation of quantitative data into “qualitized” data so that it is in the same format as the data from qualitative studies (ie, textual descriptions or narrative interpretation of the quantitative data; see below for further details); and (2) the thematic synthesis of the qualitized and quantitative data to create themes that reflect the data of included studies to answer the research questions. See Figure 1 for an outline of the process.^28^

#### Transforming Quantitative Data Into Qualitized Data

Four reviewers (S.Q., D.S., K.L., and M.C.) were involved in the transformation of quantitative data into qualitized data, using thematic analysis.^29^ First, the 4 reviewers familiarized themselves with the data by reading and re-reading all quantitative data that related to the study aims and then independently transformed them into textual descriptions (ie, text that describes quantitative data).^30^^,^^31^ Second, these descriptions were coded independently by the 4 reviewers, using thematic analysis and 1 or more of the following methods: modal (description of a group of participants around the most frequently occurring attributes), average (description of a group of participants around the mean of an attribute), comparative (description based on the comparison of participants to each other on 1 or more sets of scores), or normative (description based on the comparison of participants’ scores to the normative scores for 1 or more instruments).^32^ All 4 reviewers then came together and discussed the similarity and overall impression of each textual description, within each study, until code consensus was achieved (Suppl. Appendix 2). The codes from quantitative data (ie, qualitized data) were used with the codes from the qualitative data in thematic synthesis.

#### Qualitative Data

Two reviewers (S.Q. and K.L.) independently coded qualitative data and 1 reviewer (D.S.) read qualitative data without coding to gain an overall impression of the data (see Suppl. Appendix 3).

#### Thematic Synthesis

Integration is the combining of qualitative and qualitized data.^28^ We did this by using thematic synthesis to develop themes, between studies, from qualitized and qualitative codes created.^33^ This was performed simultaneously (convergent) rather than sequentially. First, the 3 reviewers came together to discuss the qualitative and qualitized codes, grouping codes according to topical similarity. Grouped codes were then organized into relevant integrated themes and subthemes with consensus reached among reviewers (Fig. 1; Suppl. Tab. 1). To allow importance of themes and subthemes to be understood, the number of studies identified in each theme were counted. Where the codes were not able to be grouped to make a descriptive theme, they were reported as a narrative as per the JBI Manual for Evidence Synthesis.^28^

## Results

### Flow of Studies Through the Review

**Figure 2:**
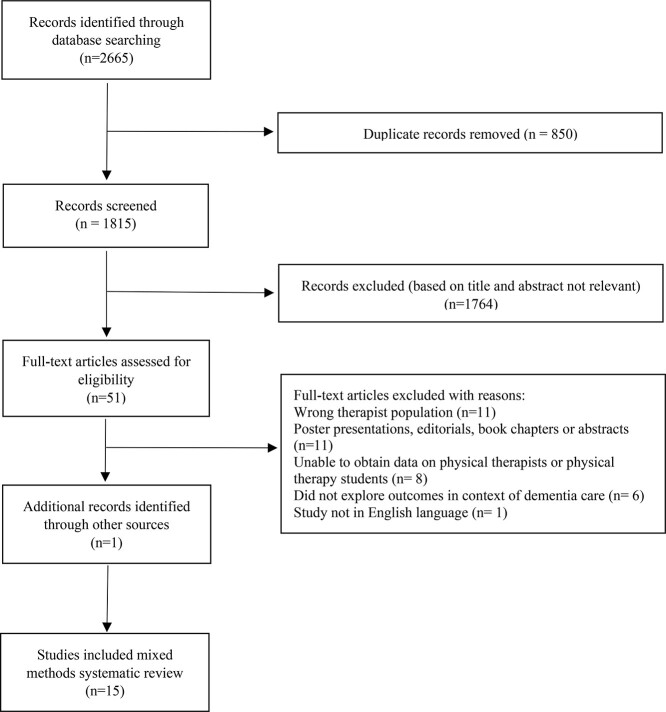
Flow of studies through the review.

The search strategy (Suppl. Appendix 1) yielded 1815 articles post removal of duplicates. After screening the title and abstract, 51 underwent full-text review. One further article included was identified following citation tracking and checking of reference lists.^34^ Thirty-seven studies were excluded after review of full texts, resulting in a final yield of 15 articles. Reasons for exclusion are outlined in Figure 2.

### Methodological Quality

The quality of all studies^24^^,^^34–47^ was rated as high (Tab. 1 and Tab. 2). Of the 9 quantitative studies included, 7 received a “yes” for all applicable items.^34^^,^^35^^,^^41^^,^^42^^,^^44^^,^^46^^,^^47^ Items around measurements of conditions, identification of confounding factors, and strategies to deal with confounding factors were not applicable to these studies, and it could not be determined in 2 studies if all outcomes were measured in a valid and reliable way, resulting in an “unclear” result for that item.^24^^,^^43^ One qualitative study received a “yes” for all applicable items.^45^ Two studies failed to meet 2 criteria,^37^^,^^40^ and 4 studies had 1 item deemed “unclear.”^36^^,^^38–40^

### Study Characteristics

The 15 studies included 1615 participants: 1496 physical therapists and 119 physical therapist students. Three studies included physical therapist students who had completed 1 clinical placement^41^^,^^43^^,^^47^ and 12 studies included physical therapists.^24^^,^^34–40^^,^^42^^,^^44–46^ Please refer to Supplementary Table 2 for more information regarding the included studies. The studies were conducted in Australia^34^ (n = 1), Europe^36–40^^,^^45^^,^^47^ (n = 7), and North America^24^^,^^35^^,^^41–44^^,^^46^ (n = 7). Quantitative data underwent data transformation (Suppl. Appendix 2) and were extracted from 9 studies.^24^^,^^34^^,^^35^^,^^41–44^^,^^46^^,^^47^ Qualitative data from 6 studies were extracted and coded (Suppl. Appendix 3).^36–40^^,^^45^ Data from all included studies^23^^,^^34–47^ underwent thematic synthesis (Suppl. Tab. 1)

We contacted the author of 1 study for missing information^35^ but were unable to obtain the required data.

### Findings of the Review

#### Themes

The thematic synthesis resulted in 7 themes (Fig. 1) relating to the research questions. There were 5 themes related to beliefs: (1) the belief that working with people with dementia is complex and challenging; (2) the belief that opportunities for education in dementia care are lacking; (3) the belief that working with people with dementia is a specialized area of practice; (4) the belief that systems are unsupportive for working with people with dementia; and (5) the belief that people with dementia deserve rehabilitation but that their potential to improve is less certain. There was 1 theme related to knowledge—lack of knowledge in some areas of dementia care—and 1 theme relating to confidence—lack of confidence working with people with dementia.

One code, desire to provide dementia care, was created from qualitized data from 3 studies^24^^,^^41^^,^^42^ but did not contribute to any themes. In these studies, physical therapists and physical therapist students reported that they did not have a preference to work with people with dementia.^41^^,^^42^

**Table 1:** Critical Appraisal Results for Included Quantitative Studies Using the Joanna Briggs Institute Critical Appraisal Checklist for Analytical Cross-Sectional Studies*^a^*

**Study**	**Q1**	**Q2**	**Q3**	**Q4**	**Q5**	**Q6**	**Q7**	**Q8**
Brody^35^	Y	Y	Y	N/A	N/A	N/A	Y	Y
Hunter^41^	Y	Y	Y	N/A	N/A	N/A	Y	Y
Hunter^42^	Y	Y	Y	N/A	N/A	N/A	Y	Y
Lawler^34^	Y	Y	Y	Y	N/A	N/A	Y	Y
Lorio^43^	Y	Y	Y	N/A	N/A	N/A	U	Y
Lusardi^44^	Y	Y	Y	N/A	N/A	N/A	Y	Y
Miles^46^	Y	Y	Y	N/A	N/A	N/A	N/A	Y
Staples^24^	Y	Y	Y	N/A	N/A	N/A	U	Y
Wood^47^	Y	Y	Y	N/A	N/A	N/A	Y	Y

^a^ N/A = not applicable; N = no; U = unclear; Y = yes. **Q1:** Were the criteria for inclusion in the sample clearly defined? **Q2:** Were the study participants and the setting described in detail? **Q3:** Was the exposure measured in a valid and reliable way? **Q4:** Were objective, standard criteria used for measurement of the condition? **Q5:** Were confounding factors identified? **Q6:** Were strategies to deal with confounding factors stated? **Q7:** Were the outcomes measured in a valid and reliable way? **Q8:** Was appropriate statistical analysis used?

**Table 2:** Critical Appraisal Results for Included Studies Using the Joanna Briggs Institute Critical Appraisal Checklist for Qualitative Research*^a^*

**Study**	**Q1**	**Q2**	**Q3**	**Q4**	**Q5**	**Q6**	**Q7**	**Q8**	**Q9**	**Q10**
Burgon^36^	Y	Y	Y	Y	Y	U	Y	Y	Y	Y
Fjellman-Wiklund^37^	Y	Y	Y	Y	Y	Y	N	Y	N	Y
Foley^38^	Y	Y	Y	Y	Y	U	Y	Y	Y	Y
Hall^39^	U	Y	Y	Y	Y	Y	Y	Y	Y	Y
Hall^40^	Y	Y	Y	Y	Y	N	N	U	Y	Y
McCarroll^45^	Y	Y	Y	Y	Y	Y	Y	Y	Y	Y

^a^ N/A = not applicable; N = no; U = unclear; Y = yes. **Q1:** Is there congruity between the stated philosophical perspective and the research methodology? **Q2:** Is there congruity between the research methodology and the research question or objectives? **Q3:** Is there congruity between the research methodology and the methods used to collect data? **Q4:** Is there congruity between the research methodology and the representation and analysis of data? **Q5:** Is there congruity between the research methodology and the interpretation of results? **Q6:** Is there a statement locating the researcher culturally or theoretically? **Q7:** Is the influence of the researcher on the research, and vice-versa, addressed? **Q8:** Are participants, and their voices, adequately represented? **Q9:** Is the research ethical according to current criteria or, for recent studies, and is there evidence of ethical approval by an appropriate body? **Q10:** Do the conclusions drawn in the research report flow from the analysis, or interpretation, of the data?

### Physical Therapists’ and Physical Therapist Students’ Beliefs on Working With People With Dementia

#### Theme: The Belief That Working With People With Dementia Is Complex and Challenging

Eight studies (physical therapists, n = 7; student, n = 1) contributed to the theme that working with people with dementia was complex and challenging.^36–42^^,^^45^ Participants believed the main challenges were communicating effectively, managing risk, behavioral symptoms, cognitive impairment, and burden on the therapist.

Participants from 2 studies believed that communicating effectively with people with dementia was complex and challenging,^38^^,^^39^ with 1 physical therapist reporting “The communication with them is something I would definitely struggle with…”.^38^^,^^39^

Participants from 3 studies believed behavioral symptoms made working with dementia complex and challenging,^37–39^ and adversely affected the ability of the person with dementia to participate in therapy.^37^^,^^38^ For some physical therapists, this was expressed as “day 1 you know they might be really agitated and they might be a little bit aggressive but another day they can be very hypo-actively delirious and you know just totally drowsy and unable to participate with physio.”^38^

A further challenge identified by physical therapists in 3 studies was providing therapy for people with cognitive impairment, including impaired memory, attention, and executive function.^38–40^ Poor memory led to what physical therapists believed to be “impaired carry-over” and in some instances feeling as though “every week it was like nearly starting all over again.”^38^

Participants in 3 studies (all physical therapists) believed managing risk was complex and challenging when working with people with dementia.^36^^,^^38^^,^^39^ Managing the risk of falling was reported as being particularly challenging.^38^^,^^39^ Physical therapists reported a tendency for people with dementia to show “risky” behaviors due to reduced insight.^36^ Participants also reported that the general principles of risk management were sometimes not applicable when caring for people with dementia. In one example, participants reported that gait aids were sometimes a risk because a person with dementia might forget the gait aid or it became “more of a trip hazard” rather than facilitating safe walking.^36^

In addition to the technical challenges of providing physical therapist care, participants from 2 studies believed that maintaining personal well-being was challenging.^41^^,^^42^ Both physical therapists and physical therapist students reported that working with people with dementia may lead to stress and burnout,^41^^,^^42^ with a low preference to work with people with late-stage dementia.^24^ Some physical therapists, however, still believed working with people with dementia to be rewarding.^42^

#### Theme: The Belief That Opportunities for Education in Dementia Care Are Lacking

Nine studies (physical therapists, n = 8; student, n = 1) contributed to the theme that there were gaps in dementia education,^24^^,^^37–42^^,^^45^^,^^46^ relating to inadequate training and the importance of learning by experience.

Participants from 8 studies believed there was inadequate training, including a lack of access to training at both an undergraduate^38^^,^^41^ and postgraduate level.^24^^,^^39^^,^^40^^,^^42^^,^^45^^,^^46^ They also reported mixed feelings about whether the training they had received was sufficient to effectively care for people with dementia,^41^^,^^42^^,^^46^ and believed they were unable to find appropriate training opportunities.^40^ They expressed a desire for further education in formats such as workshops or case studies.^38^ Participants (all physical therapists) in 3 studies expressed the importance of learning through experience due to a lack of other educational opportunities.^37–39^ Physical therapists believed they learned “on the job”^38^ through a process of “trial and error”^38^^,^^39^ and gained insight through having the “courage to try”^37^ new management strategies.

#### Theme: The Belief That Working With People With Dementia Is a Specialized Area of Practice

Six studies (all physical therapists) contributed to the theme that working with people with dementia was a specialized area of practice,^36–40^^,^^45^ requiring an understanding of the complexity of the clinical presentation, nuanced care, importance of having sufficient time, and using a holistic approach.

Participants (all physical therapists) from 3 studies believed that working with people with dementia required an understanding of the complexity of clinical presentations.^38^^,^^39^^,^^45^ They identified that people with dementia rarely presented with a diagnosis of dementia alone. In clinical settings, these individuals usually presented with other acute injuries (eg, hip fracture),^38^ comorbidities (eg, respiratory disease),^38^ or geriatric syndromes (eg, delirium)^39^ that further complicated care.

The nuanced ability to work with people with dementia was reported by physical therapists in 4 studies.^37–40^ Participants believed that dementia was often “hiding” other symptoms (eg, pain)^37^ and that the ability to identify, assess, and treat these symptoms was a specialized skill that is “hard to define”^39^ and often required establishing a connection with the person with dementia^37^ and the skilled application of non-verbal communication skills.^37^^,^^39^

In 5 studies, experienced physical therapists believed that time was important when working with people with dementia.^36–40^ For example, it was important to “have the time to read facial expressions and look at movement quality” when prescribing and administering an exercise program.^37^ Time was also seen as necessary to build rapport,^38^ facilitate learning,^36^ and manage behavioral changes.^37^

Participants (all physical therapists) from 4 studies believed that a holistic, team-based approach to care was critical to effective management.^37–39^^,^^45^ Participants identified the importance of including family as “proxy physical therapy assistants” in therapy sessions to improve engagement with physical therapy treatment, reducing anxiety and agitation.^38^ In addition to family involvement, a coordinated multidisciplinary approach to care and engagement was reported to be important for therapists, allowing for more opportunities to learn and develop skills.^38^^,^^39^

#### Theme: The Belief That Systems Are Unsupportive for Working With People With Dementia

Six studies (physical therapist, n = 6) contributed to the theme that systems are unsupportive for working with people with dementia.^24^^,^^36–39^^,^^45^ In particular, participants believed that the environment, risk aversion, and limited time were barriers in facilitating care for people with dementia.

In 5 studies, participants (all physical therapists) identified that health and aged care environments were often not conducive to care.^24^^,^^36^^,^^38^^,^^39^^,^^45^ Participants believed a familiar environment was important, with home considered the most appropriate for assessment where possible.^36^ Hospital and outpatient settings were seen as particularly detrimental for people with dementia due to “unfamiliarity.”^39^ Participants also reported that aged care facilities had features (eg, patterned carpet, lack of appropriate signage) that are known barriers to mobility in people with dementia and resulted in physical therapists feeling “disempowered.”^45^

Participants (all physical therapists) from 3 studies believed that staff in health care settings and nursing homes had a risk-averse culture, which had the potential to influence outcomes.^36^^,^^39^^,^^45^ Not allowing people to walk without a gait aid or not recommending discharge home with a higher risk of falling were examples of the difficulty faced in balancing risk and patient outcomes.^39^

The importance of time also featured as part of this theme. In 5 studies,^24^^,^^36–39^ physical therapists believed that they did not have the time required to effectively care for people with dementia. Increasing demand on services,^36^^,^^37^^,^^39^ pressure to facilitate discharge (ie, patient flow) in hospital settings,^36^^,^^37^ a perceived lack of evidence for physical therapy care,^39^ and the impact of time on therapy for people with dementia compared with other diagnoses^38^ made it difficult for physical therapists to provide effective therapy.

#### Theme: The Belief That People With Dementia Deserve Rehabilitation but That Their Potential to Improve Is Less Certain

Seven studies (physical therapists, n = 6; student, n = 1) contributed to the theme around whether people living with dementia had “rehabilitation potential.”^24^^,^^39–42^^,^^45^^,^^46^ Rehabilitation potential was discussed with reference to variability in outcomes and access to rehabilitation.

In 7 studies, physical therapists’ and physical therapist students’ beliefs on rehabilitation outcomes were variable.^24^^,^^39–42^^,^^45^^,^^46^ Physical therapists and students in 5 studies believed people with dementia would have “poor outcomes from physical therapy”^45^ and lower rehabilitation potential compared with those without dementia.^24^^,^^39^^,^^41^^,^^42^^,^^46^ However, physical therapists with experience also believed that positive outcomes were possible in this population, notably expressed in people with hip fractures.^39^^,^^40^^,^^45^

Physical therapists (n = 5) and physical therapist students (n = 1) from 6 studies discussed beliefs about access to rehabilitation.^24^^,^^39–42^^,^^46^ In 3 studies, most participants believed people with dementia deserve equality in access to care and rehabilitation.^41^^,^^42^^,^^46^ In contrast, 2 studies^39^^,^^40^ reported limited access may have been due to therapeutic nihilism. The impact of therapeutic nihilism was clearly articulated in 1 study, where physical therapists reported frustration that a “diagnosis of dementia could exclude people [with dementia] from accessing some services.”^39^ They were concerned people living with dementia were often “written off far too early,”^39^ were labelled as having “no rehabilitation potential,” and that this label is one “they can’t get rid of.”^39^ These labels were sometimes applied by other health care providers, which prevented the person with dementia from being referred to physical therapy. Sometimes this label could also be applied by their colleagues, particularly physical therapists working in acute hospital settings, who assumed the person with dementia had no rehabilitation potential and therefore did not even attempt to engage them in physical therapy.^39^^,^^40^

### Knowledge and Confidence of Physical Therapists and Physical Therapist Students in Working With People With Dementia

#### Theme: Lack of Knowledge in Some Areas of Dementia Care

Nine studies (physical therapists, n = 7; physical therapist students, n = 2) contributed to the theme of lack of knowledge in some areas of dementia care.^34^^,^^35^^,^^38–42^^,^^44^^,^^47^ Physical therapists and physical therapist students from 6 studies reported or showed a lack of general knowledge about dementia.^34^^,^^38–40^^,^^44^^,^^47^ Knowledge was lacking in the areas of behavioral management for both physical therapists^34^^,^^35^^,^^38^^,^^42^ and physical therapist students.^41^ Pain management was also a knowledge gap for physical therapists^35^^,^^42^ and physical therapist students.^41^ Understanding of communication strategies when working with people with dementia was noted to be a knowledge gap in 3 studies.^34^^,^^38^^,^^41^

#### Theme: Lack of Confidence Working With People With Dementia

Eight studies (physical therapists, n = 5; physical therapist students, n = 3)^35^^,^^39^^,^^41–43^^,^^45–47^ contributed to the theme relating to confidence in relation to working with people with dementia. In 6 studies, a lack of confidence with providing dementia care in general was observed,^35^^,^^39^^,^^43^^,^^45–47^ with this expressed as “a significant fear and panic of treating people with dementia.”^39^ Working with people with communication and/or behavioral issues was particularly linked with reduced confidence in 2 studies involving physical therapists^42^ and physical therapist students.^41^

## Discussion

This mixed-methods systematic review provides insights into the attitudes, beliefs, knowledge, and confidence of physical therapists and physical therapist students working with people with dementia. Seven themes evolved from the 6 qualitative and 9 quantitative studies included in this review: (1) the belief that working with people with dementia is complex and challenging; (2) the belief that opportunities for education in dementia care are lacking; (3) the belief that working with people with dementia is a specialized area of practice; (4) the belief that systems are unsupportive for working with people with dementia; (5) the belief that people with dementia deserve rehabilitation but that their potential to improve is less certain; (6) lack of knowledge in some areas of dementia care; and (7) lack of confidence working with people with dementia. These findings highlight a number of unmet needs.

Although physical therapists believed that positive outcomes are possible for people with dementia, they also believed that they have limited access to rehabilitation. Consistent with other health professionals,^48^ some physical therapists and students believed that this was because both their peers and other health professionals thought that people with dementia have limited rehabilitation potential (termed therapeutic nihilism in the literature).^49^ This is despite evidence that exercise can improve physical functioning and activities of daily living in people with dementia.^9^^,^^11^ Beliefs of therapeutic nihilism about the ability of people with dementia to improve may influence referral decisions for further rehabilitation and impact on quality of care.^39^^,^^48^ For example, poorer quality of care has been found for people with preexisting dementia admitted for a stroke, where they were less likely to be treated in a stroke unit or have a physical therapy assessment within 48 hours.^50^ This is despite Australian stroke guidelines recommending this for all patients irrespective of age or stroke severity.^51^ Similarly, post hip fracture, people with dementia were less likely to be referred for rehabilitation despite their ability to improve if given a longer length of stay.^52^

Negative beliefs about working with people with dementia may be due to beliefs about poor job satisfaction or a lack of knowledge of the field.^53^ In this systematic review, physical therapists and students lacked knowledge and confidence in communicating with people living with dementia as well as in managing the impact of cognition and behavior on learning and risk. Additional complexity arose when individuals presented with other co-morbidities such as a hip fracture, pain, delirium, or more severe dementia. In other health professionals, greater experience, knowledge, and levels of education were associated with more positive beliefs^54^^,^^55^ and confidence.^16^^,^^54^ However, of concern in this review, many physical therapists and physical therapist students thought that their training did not adequately prepare them for working with people with dementia.^41^^,^^42^ These findings highlight a need for greater dementia-specific education at both an under- and post-graduate level, with participants wanting more case-studies, workshops, and inter-professional learning opportunities.^38^ A few small pre–post–studies show promise in this area.^43^^,^^47^ For physical therapy and nursing students, lectures and interactive workshops followed by clinical experience in care homes improved both knowledge and confidence,^47^ and for Doctor of Physical Therapy students a multimodal program (lectures, a virtual dementia tour, communication strategies, 2 hours of clinical work, and an interactive book club) improved both confidence and empathy in working with people with dementia.^43^ After graduating, physical therapists reported benefit from learning from other health professionals, which suggests that inter-professional mentorship and supervision sessions would help consolidate knowledge from more formal training programs.^37^^,^^38^^,^^56^

Current education frameworks^57^ and dementia guidelines^58–60^ are useful for informing the content of dementia training for all health professionals, but additional physical therapy–specific training or modules may be required. There appears to be need for topics on capacity and decision-making, working with carers and family, cognitive screening tools, and how different cognitive domains can affect safety and risk.^36^^,^^38–40^^,^^45^ Interestingly, few participants mentioned differences in how people with dementia might learn new skills, with evidence that people with Alzheimer-type dementia might benefit from strategies such as errorless learning aimed at implicit rather than explicit memory.^61^ Future randomized controlled trials are needed to examine whether the above suggested content and delivery styles improve students’ and physical therapists’ knowledge and confidence as well as outcomes for people with dementia.

The call for dementia to be a specialized area of physical therapy is interesting. Many of the required strategies and skills in working with people with dementia are also useful in managing other neurological conditions such as stroke,^62^ multiple sclerosis,^63^ and traumatic brain injury.^64^ Raising the profile of working in this area may encourage more physical therapists to consider working in the field. However, the belief that working with this population would result in significant stress and burnout was apparent in a number of studies.^41^^,^^42^ Increased levels of stress and burnout have been associated with poor retention rates and job satisfaction in other health care workers^65^ and fields (eg, mental health).^66^ Debriefing with colleagues, further education, utilizing clinical supervision, and discussions with line managers are important coping strategies that could be formally implemented.^66^^,^^67^

The role of physical therapy in working with people with dementia is growing. There is increasing evidence for physical activity in addressing not only mobility, activities of daily living, and falls prevention but also in managing neuropsychiatric symptoms and in slowing cognitive decline.^8^^,^^11^^,^^68^ Physical therapists may see people with dementia in multiple settings, including the community, hospital, and residential aged care, and arguably have an important role from rehabilitation at the time of diagnosis^50^ to end of life.^65^ However, unsupportive systems and lack of knowledge about the role of physical therapy may make it hard to achieve meaningful outcomes in a complex population with a progressive condition. Governments, health services managers, and other health professionals may require further education and lobbying around physical therapists’ role in order to facilitate early referrals, fund evidence-based positions in services such as cognitive clinics, and address barriers such as time and dementia-unfriendly environments, mentioned by some participants.^38^^,^^39^^,^^45^

## Limitations

A strength of this study was the ability to capture both qualitative and quantitative studies related to the topic across several settings and countries. To combine studies, a convergent integrated approach was used following the JBI guidelines for mixed-methods systematic reviews. We published the protocol and used a robust search strategy, incorporating a number of relevant search terms and databases.

A limitation of the existing literature is the small number of papers on physical therapist students. These were of survey design rather than interviews, which did not allow for comprehensive exploration of the phenomena of interest (ie, as would semi-structured interviews). As a result, some findings are likely to be more generalizable to qualified physical therapists. Most studies were conducted after 2016, indicating a relatively new field of research despite the presence of a single article in 1994. Due to the relatively low number of studies found, we were unable to separate findings by settings (eg, community vs hospital) or level of experience. Although a number of studies used validated questionnaires,^41^^,^^42^ others were developed by authors without validation.^24^^,^^35^^,^^43^^,^^46^ Furthermore, the questionnaire used by Lusardi was developed in 1994 and contains some knowledge questions that are now outdated.^44^ A limitation of our review is that it is possible we have missed findings from studies that were not published in English or incorporated physical therapists in a wider study of health professionals. Furthermore, we were unable to clarify findings with the author of 1 study,^35^ meaning we have missed the coding of a small amount of data. Finally, we did not include gray literature or studies that were not from full-text articles, which limits our sources of data. However, including only peer-reviewed articles, all of which rated “high” based on the relevant critical appraisal checklists, increases confidence in our findings.

## Conclusions

Physical therapists and physical therapist students believe that working with people with dementia can be complex and challenging. Low levels of knowledge and confidence in areas important to working with people with dementia suggest more dementia education is needed.

## Supplementary Material

PTJ-2021-0242_R2_Supplementary_Appendix_1_pzac010

PTJ-2021-0242_R2_Supplementary_Appendix_2_pzac010

PTJ-2021-0242_R2_Supplementary_Appendix_3_pzac010

## Systematic Review Registration

This mixed-methods systematic review was prospectively registered in PROSPERO (CRD42020181845), and the protocol was published.
