# Supplementary material for: Physical Therapist and Physical Therapist Student Knowledge, Confidence, Attitudes, and Beliefs About Providing Care for People With Dementia: A Mixed-Methods Systematic Review
Source: Phys Ther. 2022 Feb 12;102(5):pzac010. doi: 10.1093/ptj/pzac010 (PMC9155993; doi:10.1093/ptj/pzac010)
Supplement: PTJ-2021-0242_R2_Supplementary_Appendix_1_pzac010 [file ptj-2021-0242_r2_supplementary_appendix_1_pzac010.pdf]

Supplementary Appendix 1. Search strategy: MEDLINE (Ovid)

1. Physical Therapists/
2. physiotherap*.mp.
3. physical therap*.mp.
4. dementia/ or exp alzheimer disease/ or exp aphasia, primary progressive/ or exp dementia, vascular/ or exp frontotemporal lobar degeneration/ or exp lewy body disease/
5. dementia.mp.
6. alzheimer*.mp.
7. Attitude/
8. Qualitative Research/
9. attitud*.mp.
10. belief*.mp.
11. confidence.mp.
12. knowledg*.mp.
13. opinion*.mp.
14. experienc*.mp.
15. reflect*.mp.
16. educat*.mp.
17. expertise.mp.
18. value*.mp.
19. perception*.mp.
20. barrier*.mp.
21. facilitat*
22. 1 or 2 or 3
23. 4 or 5 or 6
24. 7 or 8 or 9 or 10 or 11 or 12 or 13 or 14 or 15 or 16 or 17 or 18 or 19 or 20 or 21
25. 22 and 23 and 24
