# Supplementary material for: Physical Therapist and Physical Therapist Student Knowledge, Confidence, Attitudes, and Beliefs About Providing Care for People With Dementia: A Mixed-Methods Systematic Review
Source: Phys Ther. 2022 Feb 12;102(5):pzac010. doi: 10.1093/ptj/pzac010 (PMC9155993; doi:10.1093/ptj/pzac010)
Supplement: PTJ-2021-0242_R2_Supplementary_Appendix_2_pzac010 [file ptj-2021-0242_r2_supplementary_appendix_2_pzac010.pdf]

Supplementary Appendix 2. Qualitizing Table

**Study**	**Textual Description**	**Code**
Brody 2016[34]	**Knowledge**	
	For physical therapists, the average proportion of correct responses to questions testing:<br>- Their knowledge of pain in people living with dementia was 48%.<br>- Their knowledge of depression in people living with dementia was 61%.<br>- Their knowledge of neuropsychiatric symptoms in people living with dementia was 58%.<br>- Their knowledge of interventions to manage neuropsychiatric symptoms in people living with dementia was 51%.	There are gaps[*] in physical therapists' knowledge level regarding pain, depression, and neuropsychiatric symptoms, including interventions to manage these neuropsychiatric symptoms, in people with dementia.
	**Confidence**	
	For physical therapists, the average proportion of correct responses to questions testing:<br>- Their confidence in managing pain in people living with dementia was 61%.<br>- Their confidence in managing depression in people living with dementia was 46%.<br>- Their confidence in managing neuropsychiatric symptoms in people living with dementia was 54%.	Physical therapists are somewhat[**] confident in regard to pain, depression, and neuropsychiatric symptoms in people with dementia.
Hunter 2020[41]	**Knowledge**	
	Physical therapists answered 90% of questions on the Knowledge in Dementia Scale correctly.	Physical therapists are knowledgeable in most areas relating to dementia.[*]

	Thirty percent of physical therapists thought that a person with dementia was just as likely to receive pain relief as a person without dementia in a hospital setting.<br><br>Physical therapists answered 90% of questions on the Knowledge in Dementia Scale correctly.	Physical therapists are less knowledgeable regarding pain management practices for people with dementia compared to other areas of dementia knowledge.
	Twenty-three percent of physical therapists disagreed with the statement 'The history and background of people with dementia plays a significant part in their behavior.'<br><br>Physical therapists answered 90% of questions on the Knowledge in Dementia Scale correctly.	Physical therapists are less knowledgeable regarding the behavior changes in dementia compared to other areas of dementia knowledge.
	**Confidence**	
	Forty percent of physical therapists rated their ability to understand the needs of a person with dementia when they cannot communicate verbally as 3/5, with 8% rating $\geq$ 4/5.	Physical therapists feel less confident working with people with dementia who cannot communicate verbally compared to working with people with dementia in general.

	Thirty-six percent of physical therapists rated their ability to work with people who have a diagnosis of dementia a 3/5, and 45% rated their ability ≥ 4/5.<br><br>(Where a score of 1/5 = ‘not confident’, a score of 3/5 = ‘somewhat confident’ and a score of 5/5 = ‘very confident’.)	
	**Beliefs**	
	Ninety percent of physical therapists disagreed or strongly disagreed that people with dementia should not have access to inpatient rehabilitation.	Physical therapists believe people with dementia should have access to inpatient rehabilitation.
	Forty-eight percent of physical therapists agreed or strongly agreed that their expectations for a positive outcome from rehabilitation for a person with dementia are less than a person without dementia, 29% neither agreed nor disagreed.	Physical therapists believe there are lower expectations for a positive rehabilitation outcome for people with dementia compared to those without dementia.
	Fifty-three percent of physical therapists agreed or strongly agreed that it is rewarding to work with people with dementia, and 7% disagreed or strongly disagreed.	Physical therapists believe working with people with dementia is rewarding.
	Fifty-two percent of physical therapists disagreed or strongly disagreed they would prefer to work with people with dementia compared to other patient groups, and 7% agree or strongly agreed.	Physical therapists believe they do not have a preference to work with people with dementia compared to other patient groups.

	Thirty-seven percent of physical therapists thought that working with people with dementia would lead to stress and burn-out, and 30% disagreed.	Physical therapists have mixed beliefs*** regarding whether working with people with dementia leads to stress and burnout.
	Seventy percent of physical therapists agreed that knowing someone with dementia made a positive impact on how they view the need for access and providing rehabilitation services.	Physical therapists who personally know someone with dementia believe this relationship has a positive impact on how they view need for access and providing rehabilitation services.
	Forty percent of physical therapists reported that additional training in dementia care was insufficient to facilitate the effective care of people with dementia.	Physical therapists with additional training on dementia have mixed beliefs*** on whether this training was sufficient to facilitate the effective care of people with dementia.
Hunter 2020[40]	**Knowledge**	
	Eleven (out of 16) questions on the Knowledge in Dementia Scale were answered correctly by more than 85% of physical therapy students.	Physical therapy students are knowledgeable in most areas relating to dementia.*
	Twenty-nine percent of physical therapy students believed that people with dementia will eventually lose all their ability to communicate.	Physical therapy students are less knowledgeable in how the progression of dementia impacts communication compared to other areas of dementia

	Eleven (out of 16) questions on the Knowledge in Dementia Scale were answered correctly by more than 85% of physical therapy students.	knowledge.
	Twenty-two percent of physical therapy students agreed with the statement “Anger and hostility occur in dementia mostly because the “aggression” part of the brain has been affected.”<br><br>Twenty percent of physical therapy students agreed with the statement “when people with dementia walk it is usually aimless”.<br><br>Eleven (out of 16) questions on the Knowledge in Dementia Scale were answered correctly by more than 85% of physical therapy students.	Physical therapy students are less knowledgeable on neuropsychiatric symptoms and behaviors of dementia compared to other areas of dementia knowledge.
	Forty-five percent of physical therapy students did not believe that a person with dementia is less likely to receive pain relief than a person with dementia when they are in hospital.<br><br>Eleven (out of 16) questions on the Knowledge in Dementia Scale were answered correctly by more than 85% of physical therapy students.	Physical therapy students are less knowledgeable on pain management practices compared to other areas of dementia knowledge.
	**Confidence**	

	Forty-seven percent of physical therapy students rated their ability to understand the needs of a person with dementia when they cannot communicate verbally a 3/5, and 42% rated their ability ≤ 2/5.<br><br>Thirty-six percent of physical therapy students rated their ability to interact with a person with dementia when they cannot communicate verbally a 3/5, and 33% rated their ability ≤ 2/5.<br><br>Thirty-one percent of physical therapy students rated their ability to manage situations when a person with dementia becomes agitated a 3/5, and 47% rated their ability ≤ 2/5.<br><br>Forty-four percent of physical therapy students rated their ability to work with people who have a diagnosis of dementia a 3/5, and 54% rated their ability ≥ 4/5.<br><br>(A score of 1/5 = 'not confident', a score of 3/5 = 'somewhat confident' and a score of 5/5 = 'very confident'.)	Physical therapy students feel less confident working with people with dementia who cannot communicate verbally or becomes agitated compared to working with people with dementia in general.
	**Beliefs**	
	Eighty percent of physical therapy students either disagreed or strongly disagreed with the statement 'Given that people with dementia have a limited ability to learn, I believe intensive inpatient rehabilitation treatments should not be offered.'	Physical therapy student's believe people with dementia should be offered intensive inpatient rehabilitation.

	Forty-five percent of physical therapy students agreed or strongly agreed that a diagnosis of dementia negatively impacts functional recovery with rehabilitation interventions, while 24% disagreed.	Physical therapy students have mixed beliefs*** on whether a diagnosis of dementia negatively impacts functional recovery.
	Forty-seven percent of physical therapy students agreed that their expectations for a positive outcome from rehabilitation are less than a person without dementia, while 36% disagreed.	Physical therapy students have mixed beliefs*** for a positive outcome from rehabilitation.
	Six percent of physical therapy students agreed they would prefer to work with people with dementia upon graduation.	Physical therapy students believe they do not have a preference to work with people with dementia post-graduation.
	Forty-three percent of students agreed that working with people with dementia would lead to stress and burn-out for them, while 26% disagreed.	Physical therapy students have mixed beliefs*** that working with people with dementia leads to stress and burnout.
	Ninety-six percent of students, that knew somebody with dementia prior to their clinical experience, reported that knowing someone with dementia made a positive impact on how they viewed the need for access to and provision of rehabilitation to this patient group.	Physical therapists who personally know someone with dementia believe this relationship has a positive impact on how they view need for access and providing rehabilitation services.

	Fifty-three percent of physical therapy students felt the academic training in the program was sufficient to allow them to effectively and empathetically work with people with dementia.	Physical therapy students have mixed beliefs*** on whether their training was sufficient to effectively and empathetically work with people with dementia.
	Sixty percent of students identified education on pharmacological management and communication strategies as insufficient for their needs.	Physical therapy students have mixed beliefs*** on whether education on pharmacological management and communication strategies is sufficient for their needs.
Lawler 2020[33]	**Knowledge**	
	Physical therapists averaged a score of 35.3/50, which was below the threshold score for 'comprehensive' dementia knowledge (45/50).	Physical therapists have gaps in 'comprehensive' dementia knowledge.*
	Sixty-four percent of physical therapists were incorrect or unsure about whether blood vessel disease is the most common form of dementia.<br><br>Forty-six percent of physical therapists were incorrect or unsure about whether Alzheimer's Disease was the most common form of dementia.	Physical therapists have gaps in their general knowledge about dementia.*

	Sixty-four percent of physical therapists were incorrect or unsure about whether most forms of dementia generally shorten a person's life.<br><br>Sixty-four percent of physical therapists were incorrect or unsure about whether having high blood pressure increases a person's risk of developing dementia.	
	Sixty-eight percent of physical therapists were incorrect or unsure about whether the sudden onset of cognitive problems is characteristic of common forms of dementia.<br><br>Fifty-one percent of physical therapists were incorrect or unsure about whether symptoms depression can be mistaken for symptoms of dementia.	Physical therapists have knowledge gaps in the signs and symptoms of dementia.[*]
	Forty-eight percent of physical therapists were incorrect or unsure about whether uncharacteristic behaviors in a person experiencing dementia are generally a response to unmet needs.	Physical therapists have knowledge gaps regarding behavior changes in dementia.[*]
	Fifty-nine percent of physical therapists were incorrect or unsure about whether medications are the most effective way of treating behavioral symptoms of dementia.	Physical therapists have knowledge gaps regarding pharmacological management of behaviors in dementia care.[*]

	Thirty-three percent of physical therapists were incorrect or unsure about whether it is possible to communicate with a person who has advanced dementia.<br><br>Forty-eight percent of physical therapists were incorrect or unsure about whether people experiencing advanced dementia often communicate through body language.<br><br>Forty-eight percent of physical therapists were incorrect or unsure about whether it is important to correct a person with dementia when they are confused.	Physical therapists have knowledge gaps regarding communication with people with advanced dementia.*
Lorio 2017[42]	**Confidence**	
	Physical therapy students' median Confidence in Dementia Scale score was 3/5.<br><br>(A score of 1/5 = 'not confident', a score of 3/5 = 'somewhat confident' and a score of 5/5 = 'very confident'.)	Physical therapy students are somewhat confident in their management of people with dementia.**
Lusardi 1994[43]	**Knowledge**	

	Physical therapists' average score on the Alzheimer's Disease Knowledge Test was 61%.	Physical therapists have gaps in their knowledge on working with people with dementia.
Miles 2019[45]	**Confidence**	
	Forty-seven percent of physical therapists either disagreed or were neutral when asked if they had the necessary skills to effectively assess and treat persons with dementia.	Physical therapists are somewhat confident[**] they possess the necessary skills to effectively assess and treat people with dementia.
	**Beliefs**	
	Eighty-one percent of physical therapists disagreed or strongly disagreed that treatment should be limited for people with dementia.	Physical therapists believe people with dementia should not have treatment limited due to their diagnosis.
	Thirty-seven percent of physical therapists agreed that a diagnosis of dementia negatively impacts functional recovery. Forty percent disagreed, and 23% were neutral.	Physical therapists have mixed beliefs[***] on whether a diagnosis of dementia negatively impacts functional recovery.
	Sixty-five percent of physical therapists were either neutral or disagreed they received sufficient continuing education on dementia from their employers.	Physical therapists believe their employer does not provide sufficient continuing education on dementia.

Staples 2012[23]	**Beliefs**	
	"Statistical inference and large effect size measures of difference were found for PT practitioners, who reported progressively negative attitudes towards working with patients across early, middle, and late stages of dementia. The only attitudinal question not found statistically significant (p = .674) was question 32:<br><br>32. Compared to other patients, I work harder to make improvements in patients with Alzheimer's disease/dementia."	Physical therapists believe they provide similar effort when working with people with dementia across the early, middle, and late stages of dementia.
	"Content of the 11 attitudinal items showing progressively negative attitudes are as follows:<br><br>31. I believe that a diagnosis of Alzheimer's disease/dementia negatively impacts functional recovery"<br><br>33. My expectations for a positive outcome with patients with Alzheimer's disease/dementia are less."	Physical therapists believe people with late-stage dementia will have less positive outcomes than those with early or middle stage dementia.

	"Content of the 11 attitudinal items showing progressively negative attitudes are as follows:<br><br>37. Compared to other patients I prefer to work with patients with Alzheimer's disease/dementia."	Physical therapists have negative beliefs regarding their preference to work with people with late-stage dementia compared to early and mid-stage dementia (and other patients).
	"Content of the 11 attitudinal items showing progressively negative attitudes are as follows:<br><br>"36. Productivity demands limit my ability to treat patients with AD/dementia.<br><br>38. I have enough resources/time to work with patients with AD/dementia as compared to other patients.<br><br>40. Nursing is supportive of my efforts to work with patients with a diagnosis of AD/dementia."	Physical therapists believe the environment is less conducive to working with people with late-stage dementia than those with early or middle stage dementia.<br><br>Physical therapists have negative beliefs regarding their time/resources in working with late-stage dementia than those with early or middle stage dementia, compared to other patients.
	Fifty-four percent of physical therapists disagreed or strongly disagreed that they had sufficient training to manage patients with later stages of dementia.	Physical therapists believe that they are less adequately trained to work with people with symptoms of late-stage dementia compared to early-stage dementia.

	Thirteen percent of physical therapists disagreed or strongly disagreed that they had sufficient training to manage patients with early stages of dementia.	
Wood 2016[46]	**Knowledge**	
	Physical therapy students' median score on the dementia knowledge survey (specifically developed for this study) was 13/20.	Physical therapy students have gaps in their knowledge regarding dementia.*
	**Confidence**	
	When reporting confidence on a scale between 0 and 100, physical therapy students recorded an average score at baseline of 46.	Physical therapy students are somewhat confident in working with people with dementia.**

Footnote: *Knowledge – If there was no comparator we term this 'gaps in knowledge'; If scores were >80% we termed this 'knowledgeable'; **For confidence (where the main tool is a 5 point Likert scale) we use 'somewhat' confident where the mode/median/average was 3. Similarly, when the percentage correct was on, or close to, 50% (e.g., see Brody 2016) we also use this terminology. ***For beliefs we use the term 'mixed' if scores were on or around 50%.
