# Supplementary material for: Physical Therapist and Physical Therapist Student Knowledge, Confidence, Attitudes, and Beliefs About Providing Care for People With Dementia: A Mixed-Methods Systematic Review
Source: Phys Ther. 2022 Feb 12;102(5):pzac010. doi: 10.1093/ptj/pzac010 (PMC9155993; doi:10.1093/ptj/pzac010)
Supplement: PTJ-2021-0242_R2_Supplementary_Appendix_3_pzac010 [file ptj-2021-0242_r2_supplementary_appendix_3_pzac010.pdf]

Supplementary Appendix 3. Qualitative Data Extraction and Coding

Study	Direct Quotes From Study	Code
Burgon (2019)[35]	People with dementia were recognised as needing more support and more time to learn new information, and to need a tailored approach. However, limited resources meant that the available support was insufficient. Health Care Personnel reported that funds were inadequate to meet the needs of some patients:<br><br>*"Our biggest difficulty actually is getting people to the exercise group safely. And that's because we don't have funding for transport."* (Physiotherapist 1, Falls Service)	Physical therapists believe that healthcare environments are not always conducive to working with people with dementia.<br><br>Physical therapists believe that there is a lack of time and resources when working with people with dementia.
	Strength and balance exercises were believed to be helpful to prevent falls. It was, however, emphasised that:<br><br>*"It's about whether they can do that [performing an exercise] safely or not." (Physiotherapist 1, Falls Service)*	Physical therapists believe that managing risk in people with dementia is complex and challenging.
	Increasing demand on services and inadequate availability of staff resulted in pressures to assess and discharge patients quickly:<br><br>*"There obviously, (...) it is a pressure to discharge and just*	Physical therapists believe that there is a lack of time and resources available working with people with dementia.

	see new patients all the time, so, we don't get long enough, I don't think we do." (Physiotherapist 3, Falls Service)	
	People with dementia were recognised as needing more support and more time to learn new information, and to need a tailored approach.	Physical therapists believe that time is important when working with people with dementia.
	People with dementia were described as more likely to adopt risky behaviours due to impairments in insight:<br><br>*"They take risks, I think…they [people with dementia] can't reason through, like the safety…" (Physiotherapist 4, Falls Service)*	Physical therapists believe that managing risk in people with dementia is complex and challenging.
Fjellman-Wiklund (2016)[36]	*"When it comes to (physical) contact with the elderly, I've found out that I like to lay a hand on the shoulder when you talk to them, and not all elderly like it. Your really have to hold back, because…the woman I told you about…from the beginning I made a catastrophic mistake and put my hand on her shoulder and she said: "Do not touch me!". I don't know what triggered it. I kind of learned that I don't touch her until she has touched me. Just such a thing has made it work better."* - (Interview 5)	Physical therapists believe that managing behavioral symptoms when working with people with dementia is complex and challenging.

	The learning process of asking and gaining important insights was extensive and they had the *"courage to try"* and perceived it as *"a challenge"*.	Physical therapists believe that learning through experience is important.
	"…to have the time to read face expressions, and look at movement quality and see. How challenging is this exercise? Is it a good exercise and how is it performed?" (Interview 3)	Physical therapists believe that time is important when working with people with dementia.
	The PTs also prepared and planned for each training session through their own contact and interpretation of each person. Their goal for the interpretation was to prepare for training at a high level at each training session and *"not go over the border"* (acceptable limit) for each person. The interpretation was based on being *"here and now"* and they *"tuned in themselves".* The PTs paid closed attention to each person's verbal and non-verbal communication. They looked at gestures, facial expressions, body posture, if the person was sitting very still or moving around, if they seemed happy or sad and how the mood eventually shifted during the training.	The belief that physical therapists require a nuanced ability to work with people with dementia.
	The PTs tried to interpret if the people seemed interested in and interacted with other people in the training room. They were attentive to anyone expressing fatigue or if the load or intensity became too high. The PTs noticed the gaze, *"how the person looks back at you"* or if the person *"drifts away"*. The PTs had a *"strong belief"* and relied on their own interpretations.	The belief that physical therapists require a nuanced ability to work with people with dementia.

	In order to prepare for and obtain optimal conditions for each training session, the PTs felt it important to be aware of each person's physical and mental health status at all times. This was facilitated by talking both to the person her/himself and to the staff at the facility in an *"interacting way"*	Physical therapists believe that a holistic, team-based approach to care is critical to effective management of people with dementia.
	The PTs described the optimal training group as having four to six people and two PTs, in order to avoid feelings of stress, for the person training and the PTs themselves.<br><br>*"If you have four persons in a group and two leaders. . .you do have time. They have time to exercise and you can chat and you can 'meet them'. If you have more people it's more stressful, you don't get the good meeting and you can't train everything you would like to do."* - (Interview 2)	Physical therapists believe that there is a lack of time when working with people with dementia.
Foley (2020)[37]	Poor short-term memory, led to what physiotherapists described as impaired 'carry-over'. Exercise instructions frequently needed to be repeated at each treatment session. Consequently an increased amount of clinical time was required.<br><br>*"poor recall, poor carry over, every week it was like nearly starting all over again" FG2P5*	Physical therapists believe that cognitive impairment makes working with people with dementia complex and challenging.<br><br>Physical therapists believe that communicating effectively with people with dementia is complex and challenging.

		Physical therapists believe that time is important when working with people with dementia.
	Consequently, when resources were limited, people with dementia were at risk of being excluded from physiotherapy care.<br><br>*"I can see eight people in a group who are okay cognitively or I can see two people in that same time who are cognitively impaired" FG2P2*	Physical therapists believe that managing risk in people with dementia is complex and challenging.<br><br>Physical therapists believe that there is a lack of time and resources available working with people with dementia.<br><br>Physical therapists believe that cognitive impairment makes working with people with dementia complex and challenging.
	Patients' inability to actively engage with physiotherapy, because of behavioural and psychological symptoms of dementia (BPSD), was emphasized as a significant barrier to care. While this was recognized as a core symptom of the dementia itself, participants described the challenge of knowing what to do when patients did not want to engage.<br><br>*"day one you know they might be really agitated and they might be a little bit aggressive but another day they can be*	Physical therapists believe that managing behavioral symptoms when working with people with dementia is complex and challenging.<br><br>Physical therapists believe that working with people with dementia requires an understanding of the complexity of clinical presentations.

	very hypo-actively delirious and you know just totally drowsy and unable to participate with physio" FG4P2	
	…physiotherapists reported that they had received minimal structured dementia education during their undergraduate and postgraduate training.	Physical therapists believe that dementia training at an undergraduate and postgraduate level is inadequate.
	Participating physiotherapists reported that they had received minimal structured dementia education during their undergraduate and postgraduate training. Rather, they relied on informal learning from experienced peers while on clinical placements or once qualified, in the workplace.	Physical therapists believe that learning through experience is important.
	However, a physiotherapist who worked in the acute hospital setting explained that the clinical environment of both outpatient clinics and hospital wards had a detrimental effect on patients' care.<br><br>*"they could be in a six bedded ward and one day they will go to the bed and the next day they will wake up and there is different faces across the way from them" FG4P3*	Physical therapists believe that healthcare environments are not always conducive to working with people with dementia.
	Family involvement in therapy sessions was viewed as essential, though time-pressures and work commitments of family members were also acknowledged. Family presence	The belief that physical therapists require a nuanced ability to work with people with dementia.

	enhanced patient-clinician communication and also served to reduce the effects of patients' anxiety and agitation. In some instances physiotherapists described recruiting family members as proxy physiotherapy assistants.<br><br>*"I remember we had this man and whenever we went in to him to try and work he would not do anything with us but as soon as his wife was there he was grand and he would work away with us no problem" FG4P2*	
	In particular, physiotherapists described working closely with OTs, frequently undertaking joint assessments of functional abilities, mobility assessments and domiciliary reviews. In some instances there was a more formal arrangement, where physiotherapists had developed services in collaboration with their local team OT.<br><br>*"I work with an OT and we developed programmes together, and we try to bring other people in, and we do an educational programme around falls prevention and we do it week in week out" FG1P4*	Physical therapists believe that a holistic, team-based approach to care is critical to effective management of people with dementia.
	An overview of dementia itself was requested for a better understanding of the presenting features, diagnostic criteria and risk factors	Physical therapists lack knowledge in dementia care.

	"I would like to understand dementia first, and before you go into the practical issues" FG1P4<br><br>Others wanted to know more about the various subtypes of dementia, as this was frequently mentioned on referral letters to them.<br><br>*"I'd like to know more about what dementia is, what the sub-types are" FG2P3*	
	Patients' inability to actively engage with physiotherapy, because of behavioural and psychological symptoms of dementia (BPSD), was emphasized as a significant barrier to care. While this was recognized as a core symptom of the dementia itself, participants described the challenge of knowing what to do when patients did not want to engage.	Physical therapists lack knowledge in management of behaviours in dementia care.<br><br>Physical therapists believe that managing behavioral symptoms when working with people with dementia is complex and challenging.
	Physiotherapists described using one-stage simplified communication techniques in order to effectively engage with people with dementia. However, others described the	Physical therapists lack knowledge in communication strategies in dementia care.

	challenges around communication and highlighted the need for more training in this area.<br><br>*"The communication with them is something I would definitely struggle with. . . I'd like to know about other interventions like validation therapy, reminiscense, managing challenging behavior" FG1P5*	Physical therapists believe that dementia training at an undergraduate and postgraduate level is inadequate<br><br>Physical therapists believe that communicating effectively with people with dementia is complex and challenging.<br><br>Physical therapists believe that cognitive impairment makes working with people with dementia complex and challenging.
Hall (2017)[38]	Physiotherapists in all settings reported that a lot of behavioural difficulties were avoided by ensuring appropriate communication. Taking time to build a relationship was reported to be vital as well as allowing the patient to lead movement.	Physical therapists believe that communicating effectively with people with dementia is complex and challenging.
	The ability to take positive risks by the physiotherapist was reported to be associated with confidence and experience of that clinician and was learnt implicitly. This was particularly evident around issues of capacity and consent, where physiotherapists felt treating people without explicit consent was a challenge.	Physical therapists believe that managing risk in people with dementia is complex and challenging.

	The importance of non-verbal communication was reported by many of the mental health physiotherapists including the importance of body positioning and allowing the person to see their face. This helped the person communicate, but also was felt to reduce some behavioural difficulties such as aggression.	Physical therapists believe that managing behavioral symptoms when working with people with dementia is complex and challenging.<br><br>The belief that physical therapists require a nuanced ability to work with people with dementia.
	…the majority of physiotherapists described pragmatic approaches to overcome memory problems. These techniques revolved around frequently prompting patients and adapting quantity and frequency of treatments.<br><br>*"they couldn't sustain any effort for very long anyway but to get them three, four, five times a day rather than one twenty minute session made much more sense"* (In-patient, physical health)	Physical therapists believe that cognitive impairment makes working with people with dementia complex and challenging.
	All participants reported a significant lack of undergraduate education around dementia, instead relying on post-graduate experience.	Physical therapists believe that dementia training at an undergraduate and postgraduate level is inadequate.<br><br>Physical therapists believe that learning through experience is important.

	The importance of clinician's experience was discussed to varying extents. There were different methods of gaining experience, with the majority of physiotherapists relying on experiential or implicit learning, with only small amounts learnt explicitly…<br><br>*"Experience! Trial and error! Did I learn about dementia during my undergraduate training? No!"* (Community, mental health)	Physical therapists believe that learning through experience is important.
	The majority of physiotherapists also reported the importance of the environment on the patients' physiotherapy, with a variety of opinions; however, it was commonly felt that a familiar environment (typically home) was the most suitable.<br><br>*"I think some are patients being assessed in the wrong environment as if they are in a very unfamiliar situation and environment, then perhaps you don't see the potential"* (Out-patient, physical health)	Physical therapists believe that healthcare environments are not always conducive to working with people with dementia.
	Physiotherapy involves challenging a person's physical ability and this, by its very nature, increases the risk of further physical injury. Reported risks included allowing a person to walk without a walking aid, or allowing them to return home with a high risk of falls. However, the extent to which a	Physical therapists believe that managing risk in people with dementia is complex and challenging.

	physiotherapist is prepared to accept this risk was suggested to affect the person's potential to improve. It was felt that some physiotherapists are reluctant to take any risks.<br><br>*"I think people are frightened of litigation and getting into trouble for things"* (In-patient, mental health)	Physical therapists believe there is an aversion of risk in healthcare settings that has the potential to influence outcomes.
	It was described in the acute setting that patients often have their mobility deliberately restricted to try and prevent the risk of further falls. This was in the form of using bed rails, sedatives or discouraging people trying to stand or walk. However, physiotherapists recognised that this was disabling people further, made physiotherapy progress slower and increased risks such as chest infections, pressure sores and cardiovascular complications. Therefore their aim was to promote mobility, while accepting that imperfect treatments were sometimes necessary. Community and mental health physiotherapists felt that avoiding risk limited the person's ability to live a fulfilled life.	Physical therapists believe there is an aversion of risk in healthcare settings that has the potential to influence outcomes.
	"I believe everyone has rehab potential if you are skilled enough, have enough time and resources to be able to objectively work on a person's goal." (in-patient, mental health)	Physical therapists believe that positive outcomes are possible in people with dementia.

	Adapting verbal communication was suggested such as breaking down instructions, using short sentences and speaking more slowly. The importance of not overloading a person with verbal input was also reported by several physiotherapists.<br><br>*I think we're all for wanting to give people as much information as they need and overwhelming them sometimes with information and some of the time it's about taking a step back."* (In-patient, mental health)	The belief that physical therapists require a nuanced ability to work with people with dementia.
	**…** the majority of physiotherapists described pragmatic approaches to overcome memory problems. These techniques revolved around frequently prompting patients and adapting quantity and frequency of treatments.<br><br>*"they couldn't sustain any effort for very long anyway but to get them three, four, five times a day rather than one twenty minute session made much more sense"* (In-patient, physical health)	Physical therapists believe that time is important when working with people with dementia.<br><br>The belief that physical therapists require a nuanced ability to work with people with dementia.
	Moreover, the differing techniques used have been suggested to increase holistic approaches to treatment and increase creativity. These skills were reportedly directly transferable to other areas of physiotherapy. This was seen as being important	Physical therapists believe that a holistic, team-based approach to care is critical to effective management of people with dementia.

	for junior staff that may be rotating through a variety of different specialities.	
	Determining potential was a challenge reported by all physiotherapists, with the term “rehabilitation potential” frequently being used in this context. This is a label that was reported to be used mainly in the acute setting, to classify whether somebody has the potential to improve physically. There was significant disagreement about the value of this label.<br><br>Generally in-patient physiotherapists found this label useful as it helped determine the patient’s pathway, although they recognised that it was often poorly used and needed to be justified. Physiotherapists working in mental health and community settings viewed this term less favourably, reporting that it was often applied to a patient too soon and could be very detrimental to future services that were offered to that patient.	Physical therapists believe that people with dementia are often deemed to have limited rehabilitation potential.
	Furthermore, it was suggested that such potential was often prejudged; assuming people with dementia could not be rehabilitated and therefore not even attempting to engage them in physiotherapy.	Physical therapists believe that people with dementia are often deemed to have limited rehabilitation potential.

	"They'd looked at her page and before I'd even questioned them about why they'd not got her out of bed they'd already said she'd got no rehab potential." (in-patient, mental health)	Physical therapists believe that people with dementia have limited access to rehabilitation.
	Several of the physiotherapists suggested that less experienced physiotherapists demonstrated a significant fear and panic of treating people with dementia *"they have a fear of dementia so I think they think "panic" and they don't understand"* (acute, mental health).	Physical therapists lack confidence providing care for people with dementia.
	Despite being one of the most prevalent symptoms of dementia, interestingly very few physiotherapists suggested using any specific strategies to overcome memory difficulties.	Physical therapists lack knowledge in dementia care.
	Further medical issues such as nutritional needs of the patient were described as being vital to ensure physiotherapy can be undertaken, however it was felt that frequently the importance of this was overlooked. Post-operative delirium was reported to hinder the acute physiotherapy and was frequently poorly managed or diagnosed in people with dementia.	Physical therapists believe that working with people with dementia requires an understanding of the complexity of clinical presentations.
	Low physiotherapy staffing levels was reported frequently. The use of physiotherapy assistants, which were recognised to be a more cost effective approach to treating this population, was adopted variably.	Physical therapists believe that there is a lack of resources for providing dementia care.

	It was also reported by several of the participants that the presence of dementia was as often being an excuse not to treat the patient or as a cause of poor outcomes.<br><br>"*its always put as a limiting factor "ooh, they're doing ok, but they have got dementia so they won't go much further" or …. "they're not doing very well, it's 'cos they've got dementia.*" (community, physical health)	Physical therapists believe that people with dementia are often deemed to have limited rehabilitation potential.
	People with dementia were described as regularly being "*written off far too early*" (community, physical health) after suffering a hip fracture, frequently without valid reason, but potentially the result of lack of knowledge or experience of treating people with dementia rather than for true physiological reasons.<br><br>Community based physiotherapists felt that patients were often judged for their potential to improve in an acute setting which was an inappropriate setting to provide physiotherapy. This often prevented them being referred to community based services. Acute physiotherapists also acknowledged the acute setting as being inappropriate for this population, but felt there was no option as they were frequently unable to refer to community based services.	Physical therapists believe that people with dementia are often deemed to have limited rehabilitation potential.

	A further frustration reported was a lack of availability of services for people with dementia. It was reported that a diagnosis of dementia could exclude people from accessing some services. *'so do they 'qualify'* for an intermediate care bed if they [are deemed to] have no rehab potential?" (Community, physical health)	Physical therapists believe that people with dementia are often deemed to have limited rehabilitation potential.<br><br>Physical therapists believe that people with dementia have limited access to rehabilitation.
	However, a concerning suggestion made by community based physiotherapists was that often people with dementia and hip fracture are not referred for ongoing physiotherapy following discharge from acute settings. This was supported by acute physiotherapists highlighting difficulty referring to acute services, or referrals getting lost in complicated referral processes. Physiotherapists in the community reported being aware of people not being referred for ongoing input with some reporting actively seeking out patients themselves.<br><br>"[he] wasn't referred to me, I came across [him] shall we say. I'd heard stories….." [Community, physical health]	Physical therapists believe that people with dementia are often deemed to have limited rehabilitation potential.<br><br>Physical therapists believe that people with dementia have limited access to rehabilitation.
Hall (2019)[39]	The use of functional exercises with visual cues was reported to be of benefit, however the person still needed verbal prompting to look at the exercise sheets.	Physical therapists believe that cognitive impairment makes working with people with dementia complex and challenging.

		The belief that physical therapists require a nuanced ability to work with people with dementia.
	'We know that we should be working with these patients, just because they have dementia doesn't mean we shouldn't work with them, but actually, practically how do you do it?'	Physical therapists believe that cognitive impairment makes working with people with dementia complex and challenging.
	There was a universal lack of education regarding dementia reported by the participants. Several had sought extra education around treating people with dementia but had been unable to find any appropriate training. The only training received was the standard electronic learning resources that their organisation provided to all staff members. This was not specific to physiotherapy and was described as being insufficient.	Physical therapists believe that dementia training at an undergraduate and postgraduate level is inadequate.
	It was felt that in view of the current lack of supporting evidence, investing a lot of time and resources into people with dementia and hip fracture was difficult to justify.	Physical therapists believe that people with dementia are often deemed to have limited rehabilitation potential.
	The major difficulty described by therapists treating people with dementia related to ensuring adherence to physiotherapy. This was frequently cited to be due to the person's memory difficulties rather than their unwillingness to take part in actual rehabilitation. The use of functional exercises with visual cues	The belief that physical therapists require a nuanced ability to work with people with dementia.

	was reported to be of benefit, however the person still needed verbal prompting to look at the exercise sheets, further emphasising the importance of having a carer or relative to assist with the exercise programme.	Physical therapists believe that cognitive impairment makes working with people with dementia complex and challenging.
	For people with dementia, who may take longer to progress with rehabilitation, this excluded them from being given a fair opportunity to improve physically…	Physical therapists believe that time is important when working with people with dementia.
	Lack of progress during the acute stage of rehabilitation was suggested to lead people to be deemed to have 'no rehabilitation potential'. This label would then prevent them from being referred for ongoing physiotherapy in the community<br><br>*'... maybe when someone with dementia gets hip fracture ... it's just about can we get them home and get them safe and not really the rehab element is totally lost... and forgotten ...there's that horrible phrase – "they've got no rehab potential"'* (PA1)	Physical therapists believe that people with dementia are often deemed to have limited rehabilitation potential.
	For people with dementia, who may take longer to progress with rehabilitation, this excluded them from being given a fair opportunity to improve physically. The lack of opportunity to undertake physiotherapy led to them being given the permanent label of having no rehabilitation potential and thus restricting the future services and interventions that were available to them. Where people were deemed to have no	Physical therapists believe that people with dementia have limited access to rehabilitation.

	rehabilitation potential in the acute setting, they were reported to not be referred for ongoing therapy and therefore were discharged directly home or to nursing or residential home placements.	Physical therapists believe that people with dementia are often deemed to have limited rehabilitation potential.
	Physiotherapists described a lack of knowledge and education surrounding dementia and how to alter their practice to accommodate the cognitive problems that a patient may have.	Physical therapists lack knowledge in dementia care.<br><br>Physical therapists believe that dementia training at an undergraduate and postgraduate level is inadequate.
	It's kind of like, somebody at this end of the spectrum is going to be completely different to somebody at that end of the spectrum. And so, it is almost like a specialism, you know, to be able to kind of cover that whole spectrum really.' (PA7)	Physical therapists believe that working with people with dementia can be complex and challenging.<br><br>The belief that physical therapists require a nuanced ability to work with people with dementia.<br><br>Physical therapists' belief that working with people with dementia is a specialized area of practice

	'The phrase is used too frequently, too often, too quickly. ... but "no rehab potential" is a phrase that we hear quite a lot and yet you find that there is potential, it depends on what that individual wants or what their circumstances are, but I think it's a very poor phrase and I think it should never be used to be truthful' (PA7)	Physical therapists believe that positive outcomes are possible in people with dementia.<br><br>Physical therapists believe that people with dementia are often deemed to have limited rehabilitation potential.
	The beliefs of the healthcare professionals appeared to affect the likelihood of a person with dementia being referred for physiotherapy after hip fracture. There was the suggestion that the lack of referral for such people may have been due, in part, to historical beliefs that people with dementia could not be rehabilitated.<br><br>'There's an attitude that we can't help them because they have dementia. I don't agree with that – I'm just saying.' (PA3)	Physical therapists believe that people with dementia are often deemed to have limited rehabilitation potential.
McCarroll (2020)[44]	…participants were doubtful that physiotherapists had the confidence, experience or education to support or hone dementia specialist skills.	Physical therapists believe that dementia training at an undergraduate and postgraduate level is inadequate.<br><br>Physical therapists lack confidence providing care for people with dementia.

	However, in this context, most physiotherapy participants felt disempowered in terms of what they could influence, when they perceived that organisational level change was required:<br><br>*“unless the environment changes, no matter how mobile we get patient A…or how strong we get them, if there’s not appropriate signage and … if there’s a patterned carpet and she is scared to walk on it because of the perceptual problems ... in some ways you could say what’s the point in a physio going in and that is my feeling”* (P10, Physiotherapist, referrer and provider [specialist domiciliary 1])	Physical therapists believe the nursing home environment is not always conducive to working with people with dementia.
	Most participants emphasised that what they described as ‘the nursing home culture’ was perceived to be the main barrier to the physiotherapists’ role; the nursing home environment was depicted as activity-limiting, which participants suggested could oppose the aim of the conventional physiotherapy role around promoting mobility.	Physical therapists believe there is an aversion of risk in nursing homes that has the potential to influence outcomes.<br><br>Physical therapists believe the nursing home environment is not always conducive to working with people with dementia.
	In describing the physiotherapists’ role when treating people with dementia in nursing homes as multifaceted, three sub themes emerged: conventional role, specialist aspects and a lack of clarity about, or a blurred role.	Physical therapists believe that working with people with dementia is a specialized area of practice.

	Integral to these accounts were the concepts of adopting an individualised approach, adjusting for the resident with dementia, managing residents' mood and behaviour and aiming to maximise a resident's function and independence:<br><br>"*So yeah it's around finding out what's meaningful for that person (..) and that's a lot around finding out about their life history (..), their interests, likes, dislikes (..), what personally is going to make them tick, what they want to be involved in*" (P5, Physiotherapist, referrer and provider [specialist domiciliary 1])	Physical therapists believe that a holistic, team-based approach to care is critical to effective management of people with dementia.
	In addition to the issue of reluctance, a small number of participants were doubtful that physiotherapists had the confidence, experience or education to support or hone dementia specialist skills. They queried the impact of specialism on task boundaries between physiotherapists' and others' roles in the health and care team, mostly describing discomfort with the idea of blurring professional roles:<br><br>*"It's very difficult I just find it quite difficult knowing where we do fit"* (P9, Physiotherapist, provider [generic domiciliary])	Physical therapists lack confidence providing care for people with dementia.<br><br>Physical therapists believe that dementia training at an undergraduate and postgraduate level is inadequate

	However, they also highlighted features of the diagnosis and nature of dementia that they considered had a negative impact on the physiotherapy role. Some felt the progressing and fluctuating nature of the disease meant that referrer's expectations from physiotherapy needed to be adjusted, but that physiotherapy could offer benefit to the resident.<br><br>*"it might not be with your treatment that you're expecting 100% improvement. So you need to kind of know (the diagnosis) i.e. where are you setting your goal and how are you gonna get there"* (P4, Physiotherapist, referrer and provider [specialist domiciliary 1])	Physical therapists believe that positive outcomes are possible in people with dementia.<br><br>Physical therapists believe that people with dementia are often deemed to have limited rehabilitation potential.
	The themes presented highlight that participants described the physiotherapy role in residents with dementia in nursing homes as multifaceted, requiring the use of their conventional focus as well as the acquisition and development of specialist knowledge and skills.<br><br>"*And there's a lot more complexity and a lot more challenges"* (P4 and P5, Physiotherapists, referrers and providers [specialist domiciliary 1])	Physical therapists believe that working with people with dementia is a specialized area of practice.<br><br>Physical therapists believe that working with people with dementia requires an understanding of the complexity of clinical presentations.

	"*you kind of just have one of those moments where you are like oh my goodness this is really, really difficult; this is a horrible thing that the family and the patient are going through and it's quite hard for, well it is for me, to see someone like that and actually I can't help and I'm sorry I can't help; that can be quite difficult.*" (P9, Physiotherapist, provider [generic domiciliary])	Physical therapists believe that working with people with dementia can be complex and challenging
